# LIFE SPACE MOBILITY IS ASSOCIATED WITH DISTINCT FACTORS BELOW AND ABOVE THE AGE OF 75 YEARS

**DOI:** 10.1093/geroni/igae098.2809

**Published:** 2024-12-31

**Authors:** Samar Assadi Khalil, Rachel Kizony, Efrat Gil, Roy Tzemah-Shahar, Maayan Agmon

**Affiliations:** Tel Aviv University, Tel Aviv, HaMerkaz, Israel; University of Haifa, Haifa, Hefa, Israel; HaEmek Medical Center, Afula, HaZafon, Israel; University of Haifa, Haifa, HaZafon, Israel; University of Haifa, University of Haifa, HaZafon, Israel

## Abstract

Life-space mobility (LSM) is associated with the quality of life and well-being of older adults, and it is influenced by individual and environmental factors. However, age-related differences are ignored. This study aimed to explore which factors are associated with LSM in community-dwelling older adults from two age groups (< 75 and≥75 years). This cross-sectional study included older adults aged 65 and over, independently mobile, without neurologic conditions or dementia. LSM, the dependent variable, was assessed using the Life-Space Assessment-(LSA). Independent variables were assessed using Timed Up and Go-(TUG) for mobility, Montreal Cognitive Assessment-(MoCA) for cognition, Activities-specific Balance Confidence Scale-(ABC) for fear of falling, Hospital Anxiety and Depression Scale-(HADS) for depression, and four-functional mobility tasks for participation. Additionally, functional status and number of medications were assessed. Two separate stepwise regression models were conducted for older adults aged< 75 and≥75 years. Two-hundred forty-two older adults (28.9% men) were included [mean(SD) age: 73.7(6.4) years], of whom 40.9% were≥75 years. Results revealed that different factors were associated with LSM in each age group. For those< 75 years, participation and TUG explained 33% and 9.4% of the variance in LSM, respectively. Conversely, for the older group, ABC, MoCA, and TUG explained 33.5%, 4.1% and 5.2% of the variance in LSM, respectively. Our finding shows that distinct factors are associated with LSM in each group, particularly mobility in the younger and fear of falling in the older. By highlighting these specific factors, interventions can be tailored more effectively to enhance the quality of life and well-being of older adults.

